# A Manual of Operative Surgery

**Published:** 1892-06

**Authors:** 


A Manual of Operative Surgery. By Frederick Treves, F. R. C. S., Surgeon to and Lecturer on Anatomy at the London Hospital ; Member of the Board of Examiners of the Royal College of Surgeons. In two octavo volumes, containing 1,550 pages, with 442 illustrations, mostly original. Per set, cloth, $9.00 ; leather, $11.00. Philadelphia : Lea Brothers & Co. 1892.

The literature of surgery is being constantly enriched by additions that come with frequent—we had almost said amazing—rapidity. This may be regarded as somewhat of a necessity, for the lines of surgical teaching as well as surgical practice have been almost entirely recast within the last ten or fifteen years. The present work, as the author states in his preface, concerns itself solely with the practical aspect of treatment by operation, with the technical details of operative surgery, and with such part of the surgeon's work as comes within the limits of an handicraft. Mr. Treves has long been known as one of the most progressive English surgeons, and he has been quoted with as much frequency of late as any of the British or Continental operators. He has shown a special aptitude for technique, and has demonstrated his capabilities as a teacher and as a contributor to the periodical literature of surgery. It would seem to be very proper, therefore, that this author, who is yet in his prime so far as years, physical and mental powers are concerned, to tell us something of what he himself has learned, the methods of applying his knowledge, and the results that may be confidently expected from an intelligent application thereof.

The first volume of Treves's manual discusses the following general divisions of the subject: First, General Principles; second, Anesthetics; third, Operations upon Arteries and Nerves; fourth, Amputations; fifth, Excisions; sixth, Operations upon Bones, Joints, and Tendons. Under the first division the student and practitioner alike, will find grouped many important subjects with which they should familiarize themselves before undertaking the responsibility of operative surgery. The second subdivision, wherein the administration of anesthetics is discussed, is not second in importance to any other surgical question. We maintain that no man is competent to set himself up as an operator, unless he is thoroughly familiar with all the various phases relating to the administration of anesthetics. He must serve an apprenticeship at this before he can turn his attention to the knife. To administer an anesthetic properly, safely, and adequately, is an accomplishment which, we regret to say, only a few are willing to undertake, and a still fewer number possess.

In Treves's book will be found some excellent suggestions that deserve to be carefully studied. In regard to the selection of an anesthetic, we have always held that in suitable cases chloroform might be given, and in other suitable cases ether, but in no case is it wise to administer what is known as the A. C. E. mixture, but we find in Treves's book the following, which presents itself to us with much force:

> The selection of the anesthetic should be regulated by: (1) Condition of the patient; (2) the nature and the length of the operation about performed.
>
> In every case the chloroformist should carefully observe the kind of patient entrusted to his care; he should notice how respiration is performed, more especially whether nasal respiration is present or not, and he should invariably feel the pulse before commencing his duties. With nitrous oxide, a stethoscopic examination of the chest is necessary, unless it is obvious from an inspection of the patient, or from other indications, that some intra-thoracic affection exists. With other anesthetics, it is usually a good plan to listen to the chest; but no prolonged stethoscopic examination need be resorted to unless special indications exist.
>
> Generally speaking, nitrous oxide is the best anesthetic for very brief operations, and ether for long cases. As already mentioned, the disadvantages connected with the latter anesthetic may, to a great extent, be removed by preceding the inhalation by nitrous oxide or the A. C. E. mixture. There are, of course, many cases in which neither nitrous oxide nor ether could be given. The following table has been drawn up to show these exceptions :

| CASES SUITABLE FOR THE A. C. E. MIXTURE. | CASES SUITABLE FOR CHLOROFORM. |
|---|---|
| Infants and very young children.<br>Most patients above sixty or sixty-five years of age.<br>Extreme obesity, especially if associated with plethora.<br>Most cases of advanced cardiac disease.<br>Affections of the air passages or pleura, attended by dyspnea or cyanosis. | Prolonged nasal or oral operations, and those with the actual cautery.<br>Cases in which neither ether nor the A. C. E. mixture is well borne.<br>Labor.<br>Marked atheroma. |

After giving in detail the methods of administration of the anesthetic, and citing the chief difficulties and dangers connected with the anesthetic state, which are unusually valuable and explicit, the author passes on to Part III., in which he considers the ligature of arteries. The anatomical relations of the arteries are very clearly considered, and amply illustrated by clear and understandable cuts. Next comes Part IV., in which operations upon the nerves are described. A large portion of Volume I. is taken up with a minute description of the various amputations. This has heretofore been considered the brilliant part of surgical treatises, and it loses none of its interest in the hands of Treves, but, in the present era, bone surgery is receiving more attention than ever, and nowhere will be found more ample treatment of the subject than in the present work.

Volume II. opens with plastic surgery, which is considered in an exhaustive manner, and numerous illustrations are introduced to explain the text. Treves is particularly able in his consideration of plastic operations upon the bladder and urethra. Part IX. of the treatise is devoted to operations upon the neck, which are always full of interest to the general surgeon. Perhaps nowhere is our author more interesting than in the section which he devotes to operations upon the abdomen. He has had a large personal experience in this department of surgery, and speaks with the knowledge of a master. His description of all operations pertaining to intestinal surgery are especially pointed, as well as his detailed account of operations upon the kidney. We have not aimed, in this cursory review, to analyze in detail all the various improvements noted in the several departments of this treatise, for it would seem unnecessary to do so, in view of the vast amount of literature which has accumulated in the various periodicals and through the Transactions of the special societies, but it has rather been our aim to point out some of the excellencies of this work of Treves, and we hesitate not to declare it to be one of the best manuals of operative surgery for the general practitioner to adopt as a guide.

In addition to the divisions of the subject that we have mentioned, the subject is further continued by dealing with surgical diseases of the rectum, head, spine, and thorax, which latter concludes the volume. A very complete index of subjects and authors is added, which makes the work one of easy reference. All through the volume, sub-heads, in display type, bring the various subjects readily before the eye of the reader, while the type and press-work are clear, and the paper of excellent quality and color. It is one of the handsomest surgical treatises that has appeared from the press in a long time.

